# Investigation and Experimental Validation of a Novel Shelter with a Paraboloid-Like Steel Roof Structure

**DOI:** 10.3390/ma19122624

**Published:** 2026-06-18

**Authors:** Jolanta Dzwierzynska, Patrycja Lechwar

**Affiliations:** Faculty of Civil and Environmental Engineering and Architecture, Rzeszow University of Technology, 35-029 Rzeszów, Poland; p.lechwar@prz.edu.pl

**Keywords:** catenary, design, grid shell, steel structure, paraboloid, experimental test, structural efficiency, curvilinear structure

## Abstract

**Highlights:**

- A discrete catenary model (DCM) as the shaping tool of an efficient grid-shell structure.
- Integrated numerical–experimental verification method.
- A paraboloid-like steel grid structure suitable for flat quadrilateral (PQ) panels.
- Efficient structures via integration of generative design and experimental research.

**Abstract:**

The decades-long development of curvilinear steel bar forms has relied on both physical and analytical modelling. This study integrates these complementary approaches to optimise the geometry and topology of a paraboloid-like steel bar structure, with the aim of enhancing structural performance and material efficiency. The developed method introduces a novel Discrete Catenary Model (DCM), generated through dynamic relaxation, to define the geometry of a steel bar roof suitable for flat quadrilateral (PQ) parallelogram panels. The DCMs were arranged in parallel at equal spacing, forming a bar grid supported by four corner columns. Static analyses were performed for various cladding materials—glass, polycarbonate, and metal sheets—to compare structural material demands, with serviceability limit states for nodal displacements and member deformations serving as key criteria. The proportions of structural material consumption for structures with glass, polycarbonate, and metal panels were 1.00:0.68:0.61. For the glass-clad variant, a physical prototype of a recreational shelter was developed and subjected to laboratory testing under near-real conditions. The test results confirmed the analytical predictions regarding the structural response under loading; the differences in nodal displacements were in the order of tenths of a millimetre. The findings indicate that the application of a parametric DCM makes it possible to obtain the intended and efficient geometry already at the preliminary design stage. Therefore, the generation of a DCM can serve as a practical tool for shaping efficient curvilinear steel bar structures with PQ panels. The proposed original method can be further developed through alternative DCM forms to design efficient steel bar roof systems.

## 1. Introduction

The design of curvilinear steel bar structures represents a dynamic and increasingly significant field in contemporary construction, integrating architectural expression with advanced structural engineering principles. Among the various curvilinear forms developed over time, structures based on catenary geometry exhibit particularly high structural efficiency.

Catenary curves have been employed in architectural and engineering applications from antiquity to the present due to their favourable response to self-weight loading. A freely suspended cable subjected exclusively to uniform gravitational loading assumes a catenary profile characterised by purely axial tensile forces. When this geometry is inverted, the catenary becomes the ideal funicular form for compression-dominated structures such as arches and vaults. In such systems, the line of thrust coincides with the structural axis, resulting in the predominant transfer of internal forces through axial compression while significantly reducing bending moments and shear forces [1,2,3].

Although the mathematical description of the catenary curve was unknown in ancient and medieval times, its structural principles were intuitively exploited by builders. Numerous arches and vaults in Roman aqueducts and Gothic cathedrals exhibit geometries that closely approximate inverted catenary shapes, enabling the efficient transfer of compressive forces. This empirical use of catenary-like forms persisted for centuries until the curve was mathematically formulated in 1691 by Leibniz, Huygens, and Johann Bernoulli. Their work marked a turning point that enabled the deliberate and analytical application of catenary geometry in structural design. Nineteenth-century advances in civil engineering and materials science facilitated the development of large-span suspension and arch bridges, with the catenary curve remaining the prevailing choice for the arch axis in contemporary long-span bridge design [4,5,6]. Building on traditional arch bridge construction principles, reference [7] applies these concepts within the framework of additive manufacturing to develop a catenary-based arch structure. In the early twentieth century, Antoni Gaudí systematically incorporated catenary geometry into his architectural practice, most notably in the Sagrada Família, where inverted hanging-chain models served as a form-finding technique. He was also among the first architects to employ hanging-rope models for the empirical development of structurally optimised designs [8,9]. Catenary geometry continues to underpin modern cable and membrane roof design, following Frei Otto’s pioneering contributions. As demonstrated in [10], catenary forms can also be effectively employed in bending-dominated lattice structures. Steel frame structures based on catenary geometry are particularly well suited for architectural pavilion applications, and reference [8] presents an innovative construction method in which the geometry of a suspended chain is fixed through welding.

Furthermore, catenary curves have been adopted as the governing funicular geometry in the form-finding and structural design of steel shell roofs, although they were often replaced by parabolic profiles due to their similar geometric and mechanical properties [11]. Reference [12] proposes a new class of ruled geometries, termed catenary-ruled surfaces, which are generated by replacing straight rulings with catenary-shaped rulings obtained through geometric transformation. In most cases, the geometry of building roof structures employing catenary curves is obtained by rotating the curve around an axis [13,14]. When the curve is rotated about its vertical axis, synclastic surfaces in the form of domes are produced. As a rule, smooth continuous catenary curves are used in shell shaping, resulting in geometrically continuous shell structures. By contrast, hanging mesh models are used to generate efficient grid-shell structures. However, in principle, such structures are not compatible with flat quadrilateral panels, which are substantially more cost-effective than curved alternatives and consequently improve the sustainability of the overall construction.

The aim of this study is to develop a method for generating efficient steel grid-shell structures that departs from the traditional use of catenary curves and hanging-mesh form-finding, introducing instead a novel generative design approach. The proposed method employs a Discrete Catenary Model (DCM), obtained from the catenary curve generated through dynamic relaxation, to define the geometry of a curvilinear steel bar dome roof suitable for cladding with flat quadrilateral (PQ) panels arranged as parallelograms.

A full-scale prototype of a recreational shelter incorporating the DCM-derived roof geometry was designed, fabricated, and experimentally evaluated. The prototype was subjected to near-real loading and boundary conditions to validate the numerical form-finding process and the structural analyses underlying the proposed methodology. The study provides a detailed examination of the computational model and the structural response of the physical prototype, followed by a comparative assessment of both datasets.

This integrated numerical–experimental framework enables systematic verification of computational accuracy and offers a robust evaluation of the structural efficiency and practical feasibility of the proposed generative design concept for steel grid-shell systems.

## 2. Designing Curvilinear Roofs with the Application of Visual Programming Tools

With the advancement of modern digital tools, structural design in architecture and construction increasingly relies on parametric and generative modelling techniques. These approaches enable precise representation of complex spatial geometries and support both topological and structural optimisation, as well as efficient material utilisation [15,16,17,18,19]. Software environments such as Revit/Dynamo [20] and Rhinoceros 3D/Grasshopper [21] facilitate the rapid generation of intricate curvilinear structures while ensuring geometric consistency—an aspect that is particularly important in the design of lightweight yet aesthetically refined roof systems. This design strategy, based on paraboloid geometry, has been applied in several studies [22,23,24]. Algorithmic design further allows for the exploration of multiple design variants and the integration of constraints imposed by prefabrication technologies and the assembly of structural system and cladding components [25,26,27]. A key advantage of generative design for curvilinear bar structures lies in the ability to easily modify and adapt the structural topology to meet design objectives. Previous research demonstrates that optimal node placement and surface subdivision can lead to favourable stress distribution, reducing local force concentrations and minimising deformation risk [28,29,30,31,32,33,34]. Selecting an appropriate topology is also critical for the potential use of flat prefabricated glass panels, including photovoltaic modules [35,36,37]. Generative modelling of curvilinear bar structures is often combined with interactive structural analysis using the Finite Element Method (FEM) through visual programming, enabling comprehensive control over the design process while accounting for both stress distribution and structural deformations [38,39,40]. The integration of generative design with FEM analysis is particularly valuable during early design stages, where rapid evaluation of structural performance under varying topologies and support conditions is essential [41,42,43,44,45,46,47,48].

Ultimately, it is crucial to validate the structural behaviour under load through experimental testing of physical models or full-scale prototypes. These tests are key to verifying design solutions and ensuring the operational safety of innovative structural systems. Over the years, such experimental studies have been conducted on steel trusses, domes, and parabolic arches, with results providing insight into actual deformations and stresses compared to computer simulations [49,50,51]. Laboratory testing helps identify areas where computational assumptions may require adjustment or further refinement [52,53]. However, due to the cost of prototype fabrication, experimental testing is often omitted. For curvilinear structures—where existing design standards do not provide clear calculation methods and the effects of external forces are difficult to predict—such testing can be critical.

The presented research integrates the use of visual programming with the experimental investigation of a structural prototype.

## 3. Shaping Approach and Geometric Model Development

A paraboloid, as a smooth synclastic surface, can be generated by translating one parabola along another, assuming the parallelism of their axes of symmetry and the perpendicularity of the planes containing these curves. A similar shaping approach was applied in modeling the roof structure of a recreational shelter. Due to the geometric similarity between the catenary and the parabola, and the use of the Discrete Catenary Model (DCM) instead of a smooth curve, the resulting roof form will be referred to as a paraboloid-like structure.

The Discrete Catenary Model (DCM) was generated as a parametric model by discretising a catenary curve obtained through a dynamic relaxation process implemented within the Rhinoceros 3D/Grasshopper environment. A particle–spring system was employed, in which an initially straight cable was discretised into finite linear spring elements. Through dynamic relaxation under prescribed loads and boundary constraints, the system converged to a state of static equilibrium. The resulting changes in spring lengths quantified the local deformations of the discrete hanging model and defined the final funicular catenary geometry.

Subsequently, the generated catenary curve was subdivided into a number of equal segments, forming the DCM. The objective of the simulation was to generate a DCM with prescribed segment lengths. The following parameters were used as control variables during the catenary simulation: total cable length, degree of cable discretisation, spring stiffness, and applied loads. An additional control parameter was the number of segments defining the DCM derived from the catenary curve.

**Figure 1:**
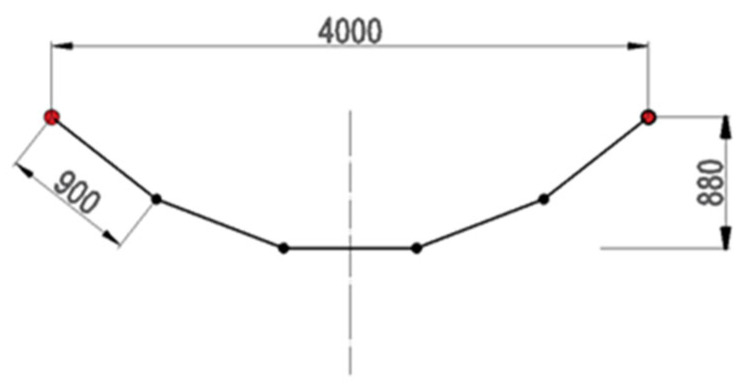
Resulted discrete catenary model (DCM).

Since the design targeted a shelter structure with plan dimensions of 4.0 × 4.0 m measured between the column axes, the distance between the cable anchor points was set to 4.0 m. The catenary curve was generated using a discretisation into ten equal segments, with a unit load applied at each node. During the simulation, the spring strength parameter was varied. The final shape of the catenary curve resulted from force equilibrium within the adopted discrete system. A subdivision of the catenary curve into five segments was established. Multiple simulations were performed, and the selection of the optimal configuration was governed primarily by the functional requirements of the roof structure. From a technological perspective, the roof panel dimensions were assumed to be within the range of 800–1000 mm. During the simulations, the shape of the DCM was continuously evaluated. A regular DCM configuration with a segment length of 900 mm was identified as a satisfactory solution, corresponding to a 12.5% increase relative to the initial segment length. Consequently, the resulting DCM formed a broken line composed of five segments, each 900 mm in length. The horizontal span of the DCM was 4000 mm, while its vertical dimension (i.e., the rise after inversion) was 880 mm, as shown in Figure 1.

The resulting DCM geometry constituted a discrete funicular configuration in static equilibrium, reflecting the bar-type structural system.

**Figure 2:**
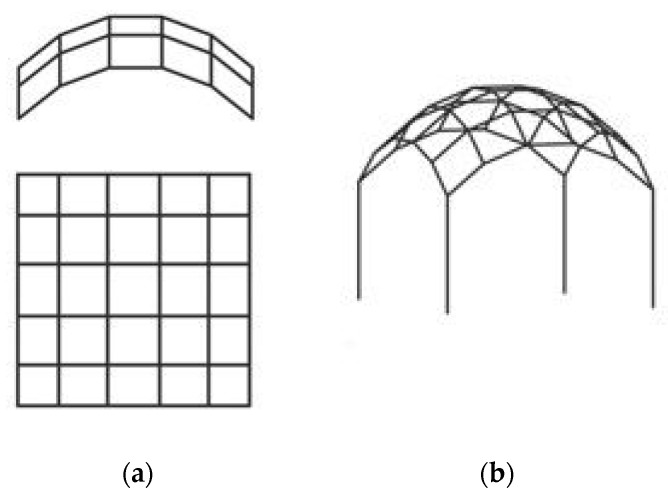
A shelter structure model: (**a**) Front and top views of the roof; (**b**) Axonometric view of the structure.

Next, the DCM generated for the given span served as a shaping curve for a paraboloid-like structure. It was translated along the generated catenary curve lying in a perpendicular plane to the DCM, while maintaining the parallelism of the corresponding vertical curve axes. DCM arches, arranged in parallel planes, were spaced at five equal intervals. Finally, straight members were introduced between them, joining the DCM vertices, forming a regular grid. All structural members were designed as straight elements to ensure a consistent and rational roof mesh. The roof was supported by four columns, each 2.1 m in height, located at the corners of the structure. The front and top views of the roof structure are shown in Figure 2a, while the axonometric view of the structural arrangement is provided in Figure 2b.

## 4. Preparation of the Computational Model and Its Analysis

In the subsequent step, a computational model was developed based on the previously established geometric model. Rigid supports were introduced at the bases of the columns, along with rigid connections at the nodes between individual structural members. The structure was modelled using S235 structural steel. This configuration was designed to enable the use of flat, quadrilateral panels as roof cladding, since the roof consisted of 25 flat mesh segments formed by straight bars. At the same time, it was assumed that the structure should exhibit efficient load-transfer behaviour. The conducted analyses and experimental tests were intended to confirm this assumption.

### 4.1. Applied Structural Loads and Load Cases

The structural model was subjected to static analysis using Autodesk Robot Structural Analysis Professional (ARSAP) [54]. The analysis was performed for three roof-cladding variants: glass panels, polycarbonate sheets, and metal sheets. In the following discussion, the structure with glass panels is referred to as T1, the one with polycarbonate sheets as T2, and the one with metal sheets as T3.

The unit loads of the roof cladding were assumed as follows:

- 0.25 kN/m^2^ for glass panels with a thickness of 10 mm;
- 0.02 kN/m^2^ for polycarbonate panels with a thickness of 10 mm;
- 0.08 kN/m^2^ for metal sheets of thickness of 1 mm.

It was assumed that the structure would be installed in Rzeszow, Poland. Therefore, the environmental loads were determined for this location, classified as:

- snow-load zone III, with a characteristic snow load s_k_ = 1.2 kN/m^2^;
- wind-load zone I, with a basic wind speed v_k_ = 22.5 m/s.

**Figure 3:**
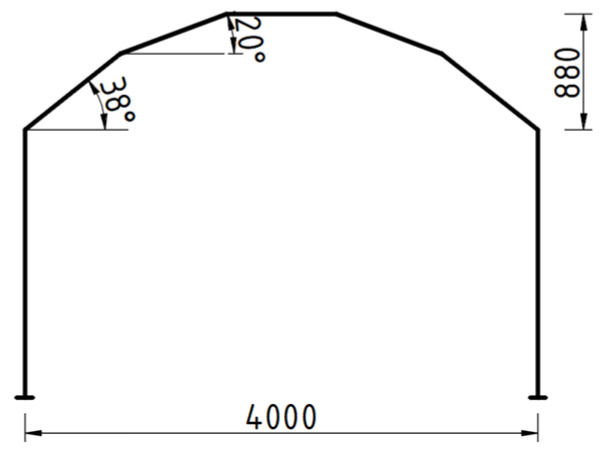
Determination of angle measures relevant to the evaluation of the roof-shape coefficient.

Due to the non-standard roof geometry and the absence of specific code guidance for calculating snow load on such a form, the roof-shape coefficient was determined in accordance with the provisions for cylindrical surfaces [55]. This approach constitutes a certain simplification. However, due to certain similarities (in terms of shape and symmetry) as well as the simplicity of the structure, this approach was adopted. Given the structural geometry—particularly the inclination angles of the individual roof members, all of which were less than 60° as indicated in Figure 3—a roof-shape coefficient 0.8 was adopted for a uniform snow distribution, whereas for an uneven distribution it had a maximum value equal to 2.0 and 0.5 for the two respective halves of the roof [55].

**Figure 4:**
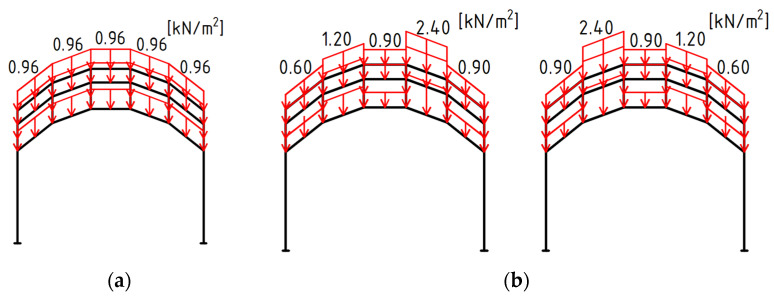
Snow load: (**a**) uniform; (**b**) uneven (right and left side of the structure).

The magnitudes of the adopted roof snow loads corresponding to uniform and non-uniform distributions are presented in Figure 4a and Figure 4b respectively.

**Figure 5:**
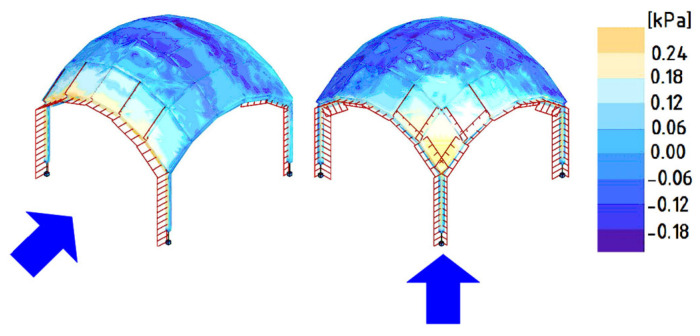
Analysed wind-load directions.

During the analysis, wind loading was automatically applied in ARSAP using numerical simulation tools, in accordance with the designated wind zone. Owing to the structural symmetry, wind action was considered in two directions, as shown in Figure 5. Both wind press and uplift forces were considered. Standard design load combinations were generated automatically in compliance with the applicable design codes [56].

To optimise structural sizing and standardise the cross-sections of members with similar functions, the bars were categorised into four groups: columns, frames, rafters, and purlins. The design process incorporated both the Ultimate Limit States (ULS) and Serviceability Limit States (SLS). Permissible deflections and node displacements were defined according to the selected roof-cladding material, reflecting the constraints imposed by feasible installation technologies. For metal-sheet cladding (structure T3), the maximum permissible node displacement was set to L/200, where L denotes the structural span. It corresponded to 20 mm in this case. For polycarbonate panels (structure T2), the limit was 10 mm (due to technological constraints resulting from the panel connection), whereas for glass panels (structure T1), the displacement was restricted to 5 mm to prevent misalignment or shifting between adjacent panels. For glass panels, the inter-panel gaps were assumed to be filled with silicone sealant. Relative displacements between adjacent glass panes exceeding 5 mm could lead to a loss of roof watertightness.

### 4.2. Results of Static Analysis for the Individual Structural Variants: T1, T2, and T3

Considering the specified design conditions, a first-order static analysis was performed to determine the structural sizing, resulting in different cross-sections and stress levels in the bars for each structural variant (T1, T2, and T3), as presented in Table 1. The calculations were carried out for closed profiles, specifically rectangular hollow sections (RHS) and square hollow sections (SHS). The decisive criterion for sizing all structural elements was compliance with the Serviceability Limit States (SLS), concerning the maximum permissible deflections and nodal displacements, as specified in Section 4.1. The obtained results are summarised in Table 1.

The observed differences in structural utilisation presented in Table 1 were caused by the different allowable deflection limits specified for structures T1, T2, and T3. However, the adoption of different cross-sectional profiles for the structural elements, corresponding to the applied roof cladding types, resulted in differences in the overall structural mass. The obtained corresponding values for various structures (T1, T2, and T3) were as follows:

- 930 kg for the structure with glass panels (T1);
- 635 kg for the structure with polycarbonate panels (T2);
- 566 kg for the structure with metal sheets (T3).

**Figure 6:**
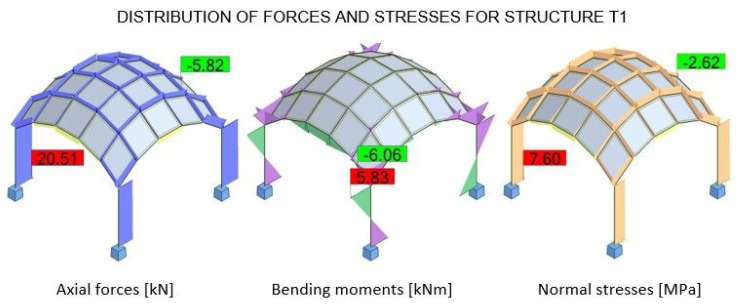
Structure T1: diagrams of axial forces, bending moments, and stresses.

**Figure 7:**
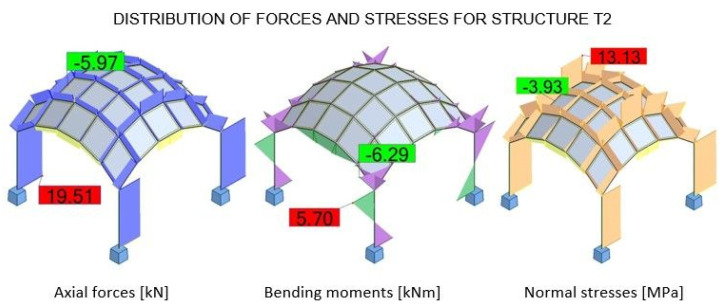
Structure T2: diagrams of axial forces, bending moments, and stresses.

**Figure 8:**
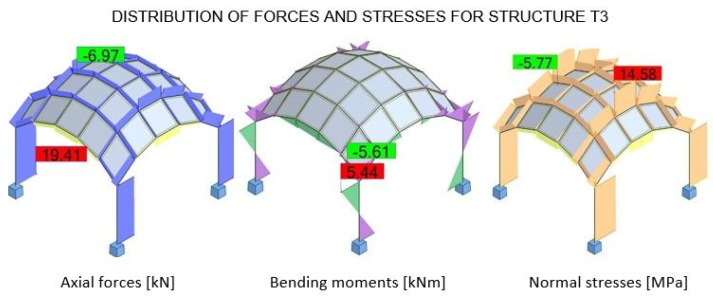
Structure T3: diagrams of axial forces, bending moments, and stresses.

The static and strength analyses enabled the assessment of internal forces acting in the bars under the applied loads. The bending-moment, axial-force, and stress diagrams for each structural variant (T1, T2, and T3) exhibited similar distributions, as illustrated in Figure 6, Figure 7 and Figure 8.

For comparative purposes, the maximum values of internal forces occurring in the structural members of variants T1, T2, and T3 were identified and are summarised in Table 2. The tabulated results represent the governing force effects obtained from the numerical analysis and provide a basis for assessing the structural response of the analysed systems.

The results presented in Table 2 indicate that the distributions of bending moments, axial forces, and stresses were similar across all structural systems. The largest bending moments occurred at the column heads. The bending moments at the roof nodes were relatively small, despite the rigid node connections. The highest axial forces were recorded in the columns for all structural variants (T1, T2, and T3), with comparable values for T1 and T2, and higher values for T3. Conversely, due to the use of larger member cross-sections in structure T1, the lowest stress levels were observed in its structural components.

## 5. Experimental Testing of the Prototype Structure

**Figure 9:**
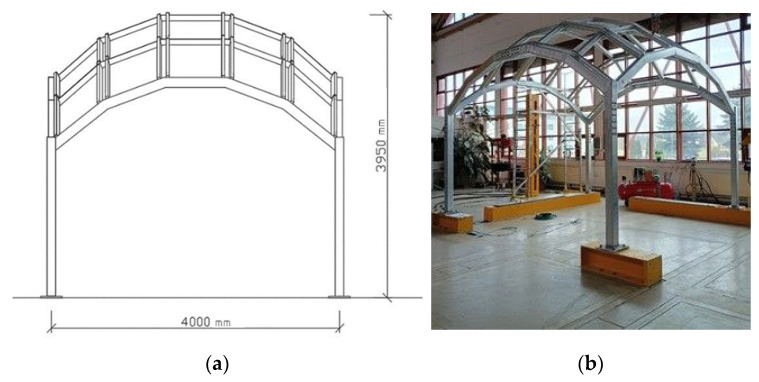
The prototype of the structure tested at the Faculty’s Structure Research Laboratory: (**a**) side view; (**b**) installation of the prototype.

For further research and analysis, structure T1 was selected. A design of this structure was developed, based on which a prototype was constructed and subsequently tested under conditions simulating real-world scenarios at the Structural Laboratory of the Faculty of Civil and Environmental Engineering and Architecture at Rzeszow University of Technology. Due to the planned point-fixed installation method for the glass panels—specifically, attaching the panes to the purlins using connectors—one purlin of type SHS 80 × 5 was replaced with two smaller-section purlins, RHS 80 × 40 × 5, as shown in Figure 9a. The roof joints were designed as welded connections, while the columns were connected to the roof using bolted joints. After the prototype components were fabricated, the structure was assembled in the laboratory and anchored to the floor elements using bolts, as illustrated in Figure 9b. During the measurements, it was monitored whether the column base anchor bolts had loosened, in order to ensure the most faithful reproduction of the assumed boundary conditions in the test model.

The structure underwent testing according to two planned schemes simulating real-world scenarios, which are described in the following sections.

### 5.1. Measurement Equipment

**Figure 10:**
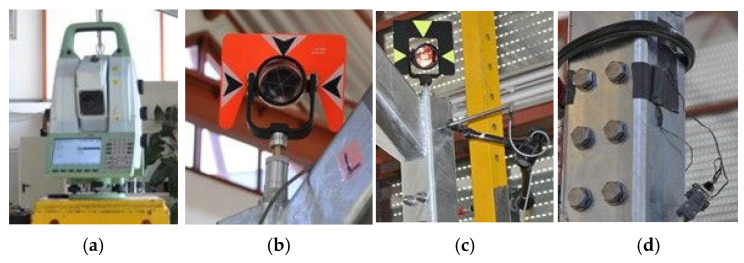
Test equipment: (**a**) a tachymeter; (**b**) a geodetic mirror; (**c**), an inductive sensor, (**d**) a strain gauge.

During the laboratory tests, the following measurement equipment was used:

- an electronic total station and geodetic mirrors for measuring vertical and horizontal displacements of the roof nodes,
- inductive sensors for measuring horizontal displacements of the columns,
- and electrical resistance strain gauges for measuring strain values in the structural bars, Figure 10.

**Figure 11:**
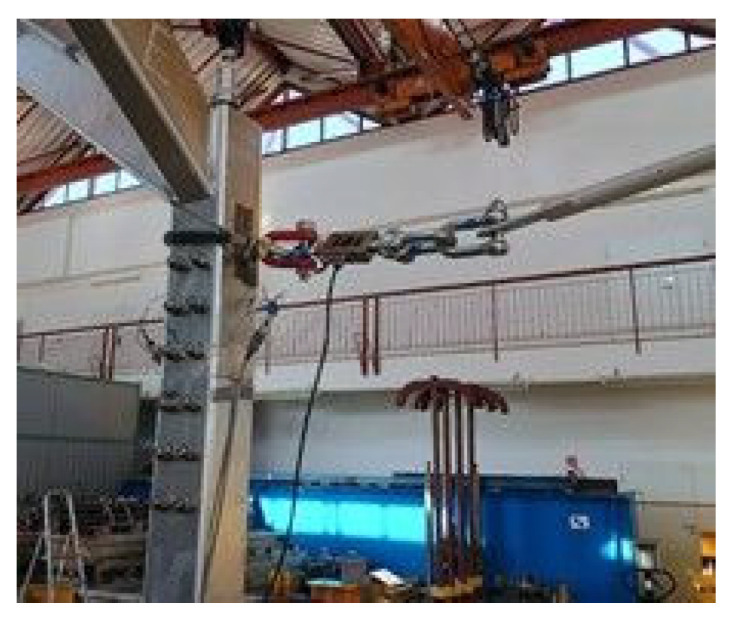
The method of mounting the turnbuckle to the structure’s column.

In the geodetic measurement method, a high-precision Leica automatic total station with a reading resolution of 0.1 mm was employed, together with geodetic prisms, as shown in Figure 10a,b. The prisms were mounted at the nodes of the roof structure, while a reference prism (working benchmark) was positioned outside the structure. Horizontal displacements of the columns were monitored using inductive displacement sensors with a 100 mm measurement base and a resolution of 0.01 mm (Figure 10c). Strain measurements were performed using electrical resistance strain gauges in combination with HBM measurement instrumentation (Figure 10d). Vertical loads were applied to the structural nodes by suspending 25 kg weights on steel cables. Horizontal forces were introduced via turnbuckles attached to the column heads. The attachment method of the turnbuckles to the structure is shown in Figure 11.

### 5.2. Laboratory Testing Procedure Based on the Developed Test Plan

The prototype of the shelter structure was subjected to laboratory testing in accordance with the developed test plan, which comprised two test schemes: Test Scheme No. 1 and Test Scheme No. 2.

#### 5.2.1. Implementation of Test Scheme No. 1

**Figure 12:**
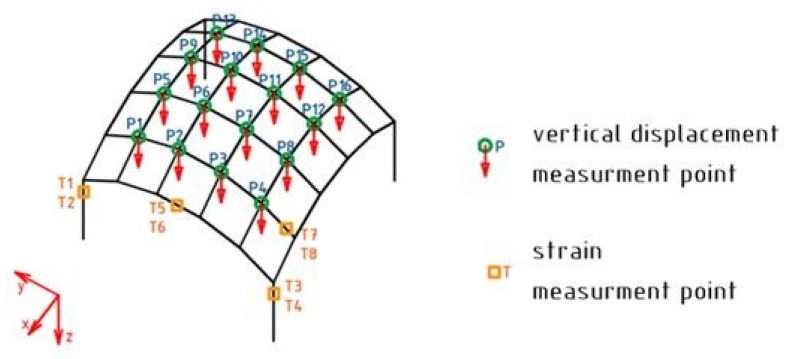
Test Scheme No. 1.

Experimental Test Scheme No. 1 was designed to replicate structural loading conditions corresponding to the weight of glass panels, snow accumulation, and the potential presence of an installer. It considered only vertical loads applied at the roof structure nodes P1–P16, which were progressively increased to the following values: 0.50, 0.75, 1.00, 1.25, and 1.50 kN per node (Figure 12).

**Figure 13:**
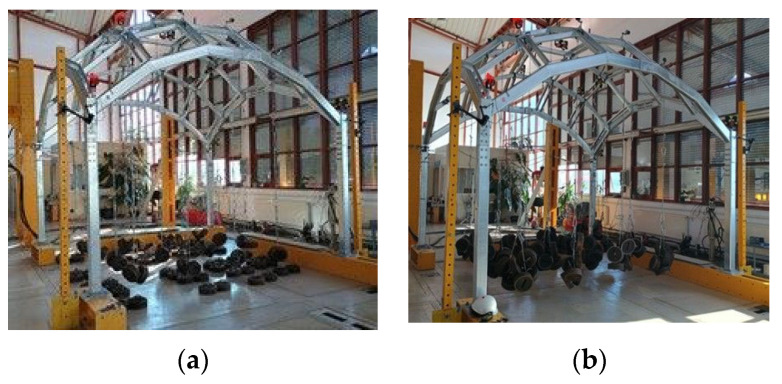
Vertical loading of the structural nodes corresponding to the test groups: (**a**) G0.50; (**b**) G1.50.

The experimental procedure comprised five test groups, denoted as G0.50, G0.75, G 1.00, G1.25, and G1.50, each corresponding to a distinct global load level applied simultaneously to 16 nodes of the roof structure, Figure 12. The spatial distribution of the applied loads and the structural configuration remained unchanged across all groups, while only the load magnitude was varied. For each group, nodal displacements and strains were recorded at four measurement stages: immediately after load application (L0), after a 5 min holding period under constant load (L5), immediately after complete unloading (U0), and after a subsequent 5 min zero-load period (U5). Displacements were measured at all 16 simultaneously loaded nodes, while strains were recorded at 8 distinct locations on the structural members, independent of the loaded nodes, Figure 12. These measurement layouts were maintained consistently across all test groups. The experimental procedure illustrated partly in Figure 13 is summarised in Table 3.

The testing procedure commenced with recording displacements under zero-load conditions PRE. During the tests, the structure was unloaded by removing the weights in a sequence that ensured symmetrical load removal. The first and the last stage of applied vertical loads with varying magnitudes are illustrated in Figure 13.

Vertical displacement was quantified at displacement points P1–P16, and structural deformation was assessed at strain points T1–T8. The complete dataset of displacement values corresponding to each measurement stage for all roof displacement points is provided in Table 4. Displacement readings were taken after applying the load and again after five minutes, as well as immediately after unloading and five minutes after unloading (L0, L5, U0, U5).

Displacement measurements at POST 0, POST 5, and POST 10 were recorded at five-minute intervals during the post-unloading phase (Table 4). Structural stresses were derived from strain gauge data using a Young’s modulus of 210 GPa for S235 steel. Table 5 summarises the resulting stress values for all measurement points.

#### 5.2.2. Implementation of Test Scheme No. 2

**Figure 14:**
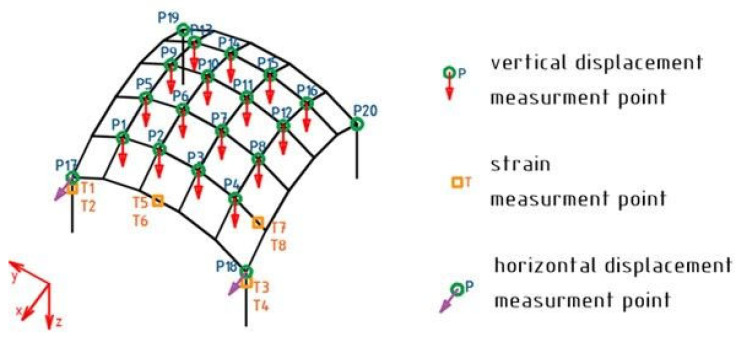
Test Scheme No. 2.

Test Sheme No. 2 was designed to simulate structural loading conditions arising from the combined effects of the glass panels’ self-weight and wind forces. A schematic representation of this loading configuration is shown in Figure 14.

Test scheme no. 2 comprised a constant vertical load applied at the roof nodes (P1–P16) in combination with a variable horizontal load introduced at two column heads (P17–P18). The vertical load of 0.25 kN per node simulated the weight of the glass panels, whereas the horizontal load was progressively increased over the course of the test. Displacement and stress readings were acquired for five horizontal force levels: 0.0 kN, 1.5 kN, 2.0 kN, 2.5 kN, and 3.0 kN. The force was measured using a strain gauge load cell, with a measurement range of up to 50 kN and a measurement accuracy of 0.01 kN.

Within this scheme, five test groups were defined: G0.25-H0.0, G0.25-H1.5, G0.25-H2.0, G0.25-H2.5, and G0.25-H3.0. Each group corresponded to a uniform vertical load applied simultaneously to 16 roof nodes, together with a horizontal load applied at two column head locations. The spatial distribution of loads and the structural configuration were kept constant across all groups; only the magnitude of the horizontal load varied. For all test groups, horizontal nodal displacements and strains were measured immediately after load application (L0). For test group G0.25-H0.00, an additional measurement (L5) was taken after a five-minute stabilisation period under constant vertical load. Displacements were measured at twenty nodes (P1–P20), while strains were recorded at eight discrete locations on the structural members (T1–T8), independently of the loaded nodes (Figure 14).

The measurement layouts were identical across all groups. The experimental procedure illustrated in Figure 14 is summarised in Table 6.

**Figure 15:**
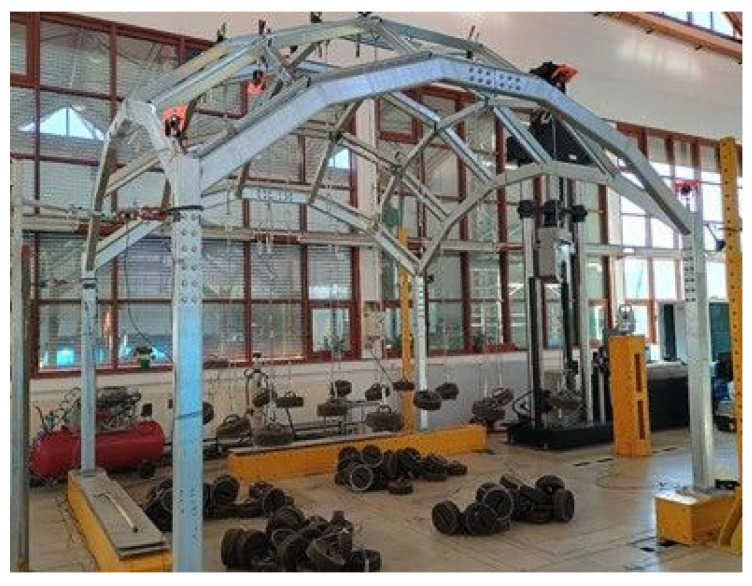
Loading of the structure during the Test Scheme No. 2 (test group G0.25-H0.00).

The structure remained under continuous load throughout the entire sequence without intermediate unloading. The loading configuration, including the application of vertical loads via calibrated weights and horizontal loads using a turnbuckle mechanism, is illustrated in Figure 15.

The values of horizontal displacement obtained during the conducted tests are summarised in Table 7. These data provide the basis for analysing the behaviour of the tested system under applied loads, allowing for an assessment of the magnitude and nature of horizontal deformations.

POST 0, POST 5, and POST 10 in Table 7 represent displacement measurements taken at designated points during the post-unloading phase of the structure, recorded at five-minute intervals. In accordance with test scheme no. 1, stresses in the structural members were calculated from strain gauge readings, assuming a Young’s modulus of 210 GPa for S235 steel. The resulting stress values for each measurement location are presented in Table 8.

## 6. Discussion

**Figure 16:**
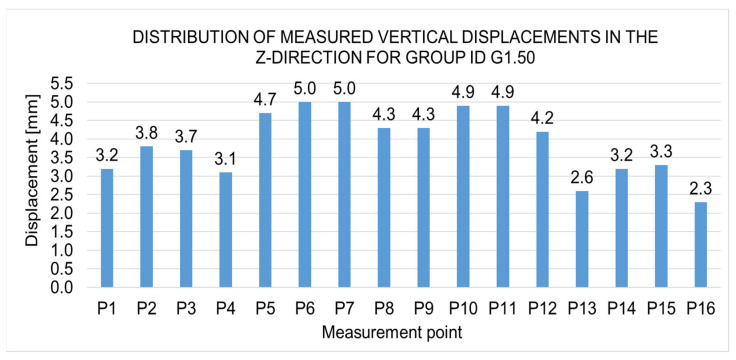
Vertical displacements in Z direction for test group G1.50.

**Figure 17:**
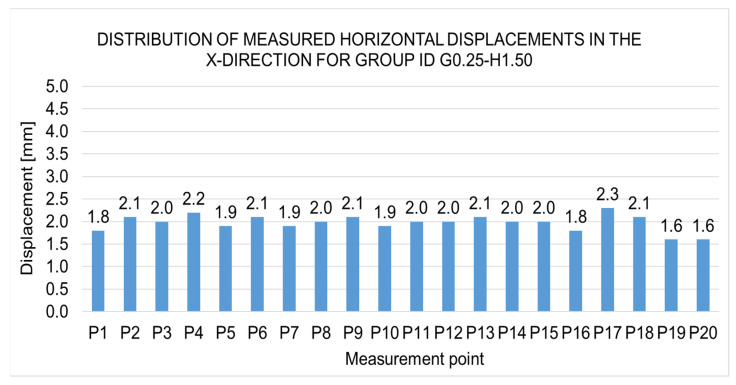
Horizontal displacements in X-direction for test group G0.25-H1.50.

The structure maintained its geometric stiffness, as it did not exhibit large displacements or signs of instability typically associated with inelastic behaviour. The largest vertical displacements of the nodes were observed at the highest points of the roof structure (P6, P7, P10, P11), but they did not exceed the allowable value of 5.0 mm, as shown in Table 4 and Figure 16. Furthermore, the displacement values of the structural nodes obtained during test schemes 1 and 2 (see Table 4 and Table 7, and Figure 16 and Figure 17) were similar for symmetrically positioned points on the roof, indicating symmetrical structural behaviour under load.

As presented in Table 2, the bending moments occurring in the roof bars were small, and the distribution of internal forces exhibited an axial character, which indicates efficient load transfer by the structure due to its geometry. Based on the conducted experimental tests and numerical analyses, it can be concluded that the experimental results correlate closely with the computational outcomes—the displacements and strain values recorded during laboratory testing did not significantly deviate from theoretical predictions. A comparison of the displacements of individual roof structure nodes calculated from the structural analysis and measured during laboratory testing is presented in Table 9. The mean discrepancy between the displacement results derived from numerical analyses and laboratory measurements was 0.5 mm.

No cracks or fractures were detected during laboratory testing, indicating that the elastic range was not exceeded and no local damage occurred. This outcome confirms a uniform distribution of internal forces, an accurate representation of the structural model, and high precision in the fabrication of structural elements and connections, which prevented the introduction of undesirable eccentricities or local weaknesses.

**Figure 18:**
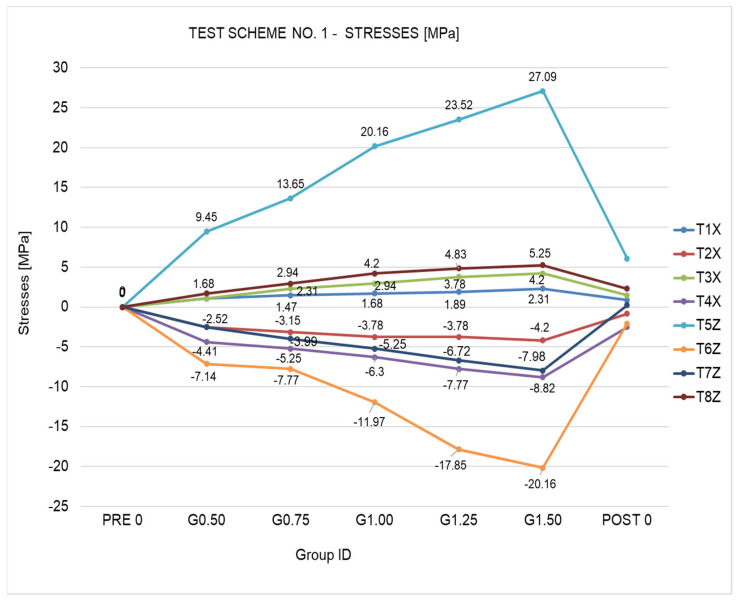
Stress values at the strain points for various test groups in Test Scheme No. 1.

**Figure 19:**
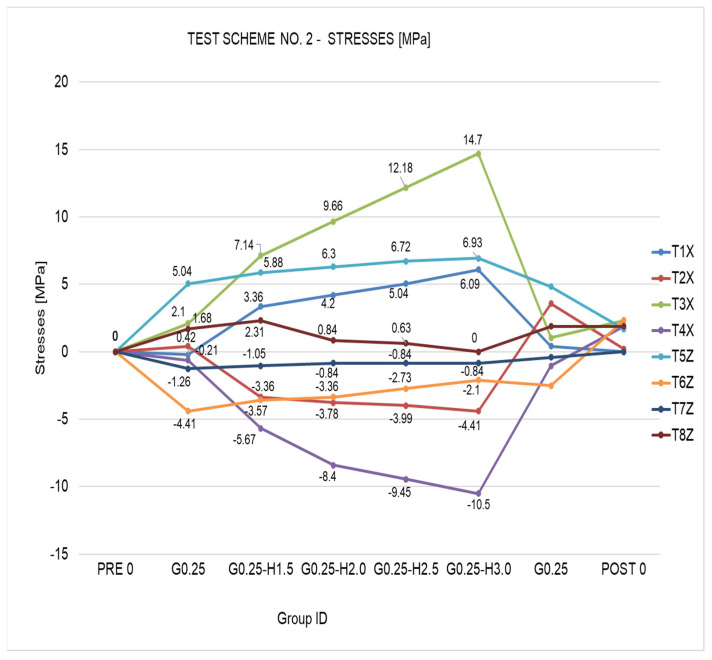
Stress values at the strain points for various test groups in Test Scheme No. 2.

Furthermore, no permanent deformations were observed; upon removal of the applied loads, the structure fully returned to its original configuration. Figure 18 illustrates the stress values at the strain points for various test groups in Test Scheme No. 1, whereas Figure 19 presents the stress values recorded for eight strain points and various test groups during Test Scheme No. 2.

The obtained results demonstrate that the stresses in all structural elements did not exceed the yield strength of the material (approximately 235 MPa for S235 steel). In addition, considering the load imposed by the glass panels and an allowable nodal displacement and strain limit of 5 mm, the structure showed a significant margin of load-bearing capacity. Since the largest displacements occurred at the top nodes of the structure—namely P6, P7, P10, and P11—and were considerably smaller at the remaining points, it is recommended that the calculated cross-sections for the top purlins be retained. However, the wall thickness of the remaining purlins may be reduced from two RHS 80 × 40 × 5 sections to two RHS 80 × 40 × 4 sections. Other dimensions should not be reduced due to technological constraints, particularly the need to maintain adequate support for the glass panels on the purlins.

The analyses also showed that if alternative roofing materials such as metal sheeting or polycarbonate panels—materials which, due to their lower stiffness, different mechanical behaviour, and method of attachment, allow for greater displacements—were used, smaller bar cross-sections could be employed, thereby reducing the overall weight of the structure. The proportions of structural material consumption for structures T1, T2, and T3 were 1.00:0.68:0.61, which significantly affects the overall construction cost. The most economical variant proved to be the canopy structure with sheet-metal cladding.

The laboratory tests confirmed the validity of the design assumptions and their compliance with the results of the static analysis. They demonstrated the favourable geometric properties of the roof formed using DCM. The internal forces in the roof bars were predominantly axial, with minimal bending moments occurring at the roof nodes. This allowed for the use of smaller cross-sections and a reduction in the amount of structural material required.

## 7. Conclusions

The article presents a novel form-finding method for shaping grid-shell roof structures based on the Discrete Catenary Model (DCM). The objective of the proposed method is to develop a structurally efficient roof form that is compatible with the use of flat (PQ) parallelogram panels. A prototype model of a recreational shelter with a roof formed using the proposed method is presented and investigated. Analytical studies supported by laboratory tests demonstrated that the DCM can serve as a practical tool for shaping efficient three-dimensional bar structures.

The parametric DCM enables the intended paraboloid-like grid-shell geometry to be achieved already at the preliminary design stage and allows for the use of flat PQ parallelogram cladding, thereby simplifying fabrication and assembly and enabling the application of various cladding materials such as polycarbonate, metal sheets, or glass, including photovoltaic (PV) panels. This is particularly advantageous in the case of glass, where flat panels are significantly more cost-effective than curved ones.

The proposed shaping method can be further developed in the context of using other DCM forms for designing efficient bar-type roof structures. However, the prototype shelter, in its current configuration or with minor modifications, can be effectively implemented in urban environments either as an aesthetically refined standalone recreational shelter or as a modular roofing system. The proposed solution contributes to advancements in contemporary structural engineering and architectural design, fostering innovative solutions for novel geometries and performance demands.

## Figures and Tables

**Table 1:** Characteristics of bars for the structures of types T1, T2, and T3.

Structure Type	Frame		Rafters		Purlins		Columns	
	Cross-Section	Utilisation	Cross-Section	Utilisation	Cross-Section	Utilisation	Cross-Section	Utilisation
	[mm]	[%]	[mm]	[%]	[mm]	[%]	[mm]	[%]
T1	RHS140 × 80 × 6	40	SHS 80 × 5	35	SHS 80 × 5	35	SHS 120 × 6	35
T2	SHS 100 × 4	64	SHS 70 × 4	74	SHS 50 × 4	67	SHS 100 × 4	76
T3	SHS 80 × 4	94	SHS 70 × 4	86	SHS 50 × 4	83	SHS 90 × 4	94

**Table 2:** The highest values of internal forces occurring in structural members.

Structure Type		T1	T2	T3
Max. bending moment [kNm]	Frame	6.06	6.29	5.61
	Rafters	1.59	1.69	1.76
	Purlins	1.0	1.08	1.09
	Columns	5.83	5.70	5.44
Max. axial force [kN]	Frame	9.67	9.49	9.83
	Rafters	5.48	6.62	5.93
	Purlins	7.03	9.44	10.53
	Columns	20.51	19.51	19.41
Max. Normal stresses [MPa]	Frame	4.36	6.25	8.19
	Rafters	3.73	6.37	5.70
	Purlins	4.78	13.13	14.64
	Columns	7.60	12.84	14.28
Displacement [mm]		4.73	9.65	20.0

**Table 3:** Experimental procedure for Test Scheme No. 1.

Group ID	Vertical Load Level [kN]	Loaded Nodes	Displacement Points	Strain Points	Measurement Stages
G0.50	0.50	P_1_-P_16_	P_1_-P_16_	T_1_-T_8_	L0, L5, U0, U5
G0.75	0.75	P_1_-P_16_	P_1_-P_16_	T_1_-T_8_	L0, L5, U0, U5
G1.00	1.00	P_1_-P_16_	P_1_-P_16_	T_1_-T_8_	L0, L5, U0, U5
G1.25	1.25	P_1_-P_16_	P_1_-P_16_	T_1_-T_8_	L0, L5, U0, U5
G1.50	1.50	P_1_-P_16_	P_1_-P_16_	T_1_-T_8_	L0, L5, U0, U5

**Table 4:** The recorded displacement values for each test group at roof displacement points P1–P16.

Group ID	Measurement Stage	P1	P2	P3	P4	P5	P6	P7	P8	P9	P10	P11	P12	P13	P14	P15	P16
		Z Direction															
		[mm]															
	PRE 0	0.0	0.0	0.0	0.0	0.0	0.0	0.0	0.0	0.0	0.0	0.0	0.0	0.0	0.0	0.0	0.0
G0.50	L0	0.9	1.2	1.0	0.9	1.2	1.5	1.5	1.2	1.3	1.5	1.4	1.2	0.7	0.9	1.0	0.7
G0.50	L5	0.9	1.3	1.1	0.9	1.3	1.5	1.5	1.3	1.3	1.5	1.5	1.2	0.8	0.8	1.0	0.7
	U0	0.1	0.1	0.0	0.1	0.2	0.1	0.2	0.2	0.2	0.1	0.1	0.2	0.0	−0.1	0.1	0.1
	U5	0.1	0.1	0.1	0.1	0.2	0.1	0.2	0.3	0.2	0.2	0.2	0.2	0.1	−0.1	0.1	0.1
G0.75	L0	1.4	1.9	1.7	1.4	2.1	2.4	2.4	2.0	2.0	2.2	2.2	2.0	1.2	1.4	1.5	1.1
G0.75	L5	1.5	1.8	1.8	1.5	2.1	2.4	2.4	2.1	2.1	2.2	2.3	2.0	1.1	1.4	1.5	1.2
	U0	0.2	0.2	0.1	0.2	0.5	0.3	0.3	0.4	0.4	0.3	0.3	0.4	0.1	0.0	0.1	0.1
	U5	0.2	0.3	0.1	0.2	0.5	0.3	0.3	0.4	0.3	0.3	0.4	0.4	0.2	0.0	0.1	0.2
G1.00	L0	2.0	2.5	2.4	1.9	3.0	3.3	3.3	2.8	2.8	3.1	3.2	2.7	1.6	2.0	2.1	1.5
G1.00	L5	2.1	2.5	2.4	2.0	3.1	3.3	3.3	2.8	2.8	3.2	3.2	2.8	1.7	2.0	2.1	1.6
	U0	0.3	0.3	0.2	0.3	0.6	0.4	0.4	0.5	0.5	0.3	0.4	0.6	0.2	−0.1	0.1	0.3
	U5	0.3	0.3	0.2	0.4	0.7	0.4	0.5	0.6	0.4	0.4	0.5	0.6	0.2	0.1	0.2	0.3
G1.25	L0	2.5	3.2	3.1	2.5	3.8	4.2	4.0	3.6	3.6	4.0	4.0	3.5	2.1	2.7	2.7	2.0
G1.25	L5	2.6	3.2	3.1	2.6	3.9	4.2	4.2	3.6	3.6	4.1	4.0	3.5	2.1	2.6	2.8	1.9
	U0	0.3	0.4	0.3	0.4	0.9	0.5	0.6	0.7	0.6	0.5	0.6	0.8	0.3	0.2	0.2	0.4
	U5	0.4	0.4	0.3	0.4	0.9	0.6	0.6	0.7	0.6	0.5	0.6	0.7	0.3	0.1	0.2	0.3
G1.50	L0	3.1	3.8	3.7	3.0	4.7	5.0	5.0	4.3	4.3	4.9	4.9	4.2	2.6	3.2	3.2	2.3
G1.50	L5	3.2	3.8	3.7	3.1	4.7	5.0	5.0	4.3	4.3	4.9	4.9	4.2	2.6	3.2	3.3	2.3
	POST 0	0.5	0.4	0.4	0.5	0.9	0.7	0.7	0.9	0.7	0.6	0.7	0.9	0.3	0.1	0.3	0.5
	POST 5	0.6	0.5	0.4	0.5	1.0	0.7	0.7	0.8	0.7	0.7	0.7	0.9	0.3	0.1	0.3	0.5
	POST 10	0.5	0.4	0.3	0.5	1.0	0.7	0.6	0.9	0.7	0.6	0.8	0.9	0.3	0.2	0.3	0.4

**Table 5:** Calculated stress values in the structural bars obtained from strain gauge measurements.

Group ID	Measurement Stage	T1X	T2X	T3X	T4X	T5Z	T6Z	T7Z	T8Z
		[MPa]							
	PRE 0	0.00	0.00	0.00	0.00	0.00	0.00	0.00	0.00
G0.50	L0	1.05	−2.52	0.84	−4.20	9.03	−7.56	−2.52	1.47
G0.50	L5	1.05	−2.52	1.05	−4.41	9.45	−7.14	−2.52	1.68
	U0	0.21	−0.21	0.00	−1.47	1.26	−0.42	0.00	0.21
	U5	0.21	−0.42	0.21	−1.26	1.05	−0.21	0.00	0.21
G0.75	L0	1.47	−3.15	2.31	−5.25	13.65	−7.77	−3.99	2.94
G0.75	L5	1.47	−3.15	2.31	−5.25	13.65	−7.77	−3.99	2.94
	U0	0.21	−0.21	0.42	−1.47	2.10	−0.84	0.00	0.84
	U5	0.42	−0.21	0.42	−1.47	2.31	−1.05	0.00	1.05
G1.00	L0	1.68	−3.57	2.73	−6.09	19.74	−11.97	−5.25	3.99
G1.00	L5	1.68	−3.78	2.94	−6.30	20.16	−11.97	−5.25	4.20
	U0	0.63	−0.42	0.84	−1.68	2.73	−1.89	0.00	1.26
	U5	0.63	−0.42	0.84	−1.68	2.73	−1.89	0.00	1.26
G1.25	L0	1.89	−3.78	3.57	−7.56	23.10	−17.85	−6.72	4.83
G1.25	L5	1.89	−3.78	3.78	−7.77	23.52	−17.85	−6.72	4.83
	U0	0.63	−0.42	1.26	−1.89	5.46	−2.73	0.00	1.89
	U5	0.63	−0.63	1.26	−2.10	5.46	−2.73	0.00	1.68
G1.50	L0	2.10	−4.20	4.20	−8.61	26.88	−19.95	−8.19	5.25
G1.50	L5	2.31	−4.20	4.20	−8.82	27.09	−20.16	−7.98	5.25
	POST 0	0.84	−0.84	1.47	−2.52	6.09	−2.10	0.21	2.31
	POST 5	0.84	−0.84	1.47	−2.52	6.09	−2.10	0.21	2.31
	POST 10	0.84	−0.84	1.47	−2.52	6.09	−2.10	0.21	2.31

**Table 6:** The recorded displacement values for each test group at roof displacement points P1–P20.

Group ID	Load Level		Loaded Nodes		Displacement Points	Strain Points	Measurement Stages
	Vertical [kN]	Horizontal [kN]	Vertical	Horizontal			
G0.25-H0.00	0.25	0.00	P_1_–P_16_	P_17_–P_18_	P_1_–P_20_	T_1_–T_8_	L0, L5
G0.25-H1.50	0.25	1.50	P_1_–P_16_	P_17_–P_18_	P_1_–P_20_	T_1_–T_8_	L0
G0.25-H2.00	0.25	2.00	P_1_–P_16_	P_17_–P_18_	P_1_–P_20_	T_1_–T_8_	L0
G0.25-H2.50	0.25	2.50	P_1_–P_16_	P_17_–P_18_	P_1_–P_20_	T_1_–T_8_	L0
G0.25-H3.00	0.25	3.00	P_1_–P_16_	P_17_–P_18_	P_1_–P_20_	T_1_–T_8_	L0

**Table 7:** Horizontal displacement results according to Test Scheme No. 2.

Group ID	Measurement Stage	P1	P2	P3	P4	P5	P6	P7	P8	P9	P10	P11	P12	P13	P14	P15	P16	P17	P18	P19	P20
		X Direction																			
		[mm]																			
	PRE 0	0.0	0.0	0.0	0.0	0.0	0.0	0.0	0.0	0.0	0.0	0.0	0.0	0.0	0.0	0.0	0.0	0.0	0.0	0.0	0.0
G0.25-H0.00	L0	0.0	0.1	0.2	0.2	0.0	0.2	0.0	0.2	0.2	−0.1	0.2	0.2	0.1	0.1	0.2	0.1	0.1	0.3	−0.1	0.0
G0.25-H0.00	L5	0.0	0.2	0.1	0.1	0.2	0.2	0.1	0.1	0.3	0.2	0.3	0.1	0.1	0.1	0.1	0.1	0.2	0.1	−0.1	−0.1
G0.25-H1.50	L0	1.8	2.1	2.0	2.2	1.9	2.1	1.9	2.0	2.1	1.9	2.0	2.0	2.1	2.0	2.0	1.8	2.3	2.1	1.6	1.6
G0.25-H2.00	L0	2.5	2.6	2.7	2.7	2.4	2.7	2.3	2.5	2.7	2.3	2.4	2.4	2.5	2.4	2.5	2.4	2.9	2.7	2.0	2.1
G0.25-H2.50	L0	3.1	3.1	3.2	3.1	3.2	3.0	3.0	3.2	3.1	3.0	3.0	3.0	3.0	3.1	3.1	3.0	3.7	3.4	2.6	2.5
G0.25-H3.00	L0	3.7	3.9	3.9	3.9	3.8	3.8	3.7	3.9	3.8	3.5	3.6	3.7	3.7	3.6	3.7	3.6	4.5	4.3	3.1	3.2
G0.25-H0.00	L0	0.3	0.5	0.6	0.6	0.4	0.6	0.5	0.7	0.6	0.5	0.5	0.6	0.3	0.5	0.6	0.4	0.5	0.6	0.1	0.3
G0.25-H0.00	L5	0.3	0.5	0.5	0.5	0.5	0.5	0.4	0.7	0.6	0.5	0.6	0.6	0.4	0.3	0.5	0.3	0.4	0.6	0.1	0.3
	POST 0	0.2	0.3	0.4	0.3	0.2	0.4	0.4	0.4	0.5	0.3	0.4	0.3	0.2	0.2	0.3	0.2	0.1	0.3	0.2	0.3
	POST 5	0.2	0.2	0.3	0.2	0.2	0.4	0.4	0.4	0.4	0.2	0.3	0.3	0.2	0.2	0.2	0.2	0.1	0.3	0.2	0.3
	POST 10	0.1	0.3	0.3	0.2	0.2	0.3	0.3	0.4	0.4	0.2	0.3	0.3	0.2	0.2	0.2	0.2	0.1	0.3	0.2	0.3

**Table 8:** Calculated stress values in the structural bars obtained from strain gauge measurements.

Group ID	Measurement Stage	T1X	T2X	T3X	T4X	T5Z	T6Z	T7Z	T8Z
		[MPa]							
	PRE	0.00	0.00	0.00	0.00	0.00	0.00	0.00	0.00
G0.25-H0.00	L0	−0.21	0.00	1.47	−0.84	4.41	−4.20	−1.47	1.26
G0.25-H0.00	L5	−0.21	0.42	2.10	−0.63	5.04	−4.41	−1.26	1.68
G0.25-H1.50	L0	3.36	−3.36	7.14	−5.67	5.88	−3.57	−1.05	2.31
G0.25-H2.00	L0	4.20	−3.78	9.66	−8.40	6.30	−3.36	−0.84	0.84
G0.25-H2.50	L0	5.04	−3.99	12.18	−9.45	6.72	−2.73	−0.84	0.63
G0.25-H3.00	L0	6.09	−4.41	14.70	−10.50	6.93	−2.10	−0.84	0.00
G0.25-H0.00	L0	0.42	3.57	1.05	−1.05	4.83	−2.52	−0.42	1.89
G0.25-H0.00	L5	0.21	3.57	1.26	−1.26	4.41	−1.26	−1.05	2.10
	POST 0	0.00	0.21	2.31	1.89	1.47	0.00	0.00	1.89
	POST 5	0.00	0.21	2.31	1.89	1.68	2.31	0.00	1.89
	POST 10	−0.21	0.21	2.31	1.68	1.68	2.52	0.00	1.26

**Table 9:** Comparison between the experimental and numerical results of point displacements.

Measurement Point	Values of Vertical Displacements in the Z Direction		
	Experimental Results	Numerical Results	Difference Value
	[mm]	[mm]	[mm]
P1	3.2	2.3	0.9
P2	3.8	3.2	0.6
P3	3.7	3.2	0.5
P4	3.1	2.3	0.8
P5	4.7	3.9	0.8
P6	5.0	4.3	0.7
P7	5.0	4.3	0.7
P8	4.3	3.9	0.4
P9	4.3	4.0	0.3
P10	4.9	4.2	0.7
P11	4.9	4.2	0.7
P12	4.2	4.0	0.2
P13	2.6	2.3	0.3
P14	3.2	3.1	0.1
P15	3.3	3.1	0.2
P16	2.3	2.3	0.0

## Data Availability

The original contributions presented in this study are included in the article. Further inquiries can be directed to the corresponding author.

## Abbreviations

The following abbreviations are used in this manuscript:

DCM	Discrete Catenary Model
PQ	Planar Quadrilateral
ARSAP	Autodesk Robot Structural Analysis Professional
ULS	Ultimate Limit States
SLS	Serviceability Limit States
BIPV	Building-integrated photovoltaics
L	the span

## References

[1] Gohnert M., Bradley R. (2020). Catenary solutions for arches and vaults. J. Archit. Eng..

[2] Nikolić D. (2019). Catenary arch of finite thickness as the optimal arch shape. Struct. Multidiscip. Optim..

[3] Block P., DeJong M., Ochsendorf J. (2006). As hangs the flexible line: Equilibrium of masonry arches. Nexus Netw. J..

[4] Wei C., Liu C., Hu Q., Han X., Wang Y., Wang L. (2025). In-plane stability behaviours of concrete-filled steel tubular catenary arches under different loading conditions. J. Constr. Steel Res..

[5] Han X., Wei C., Hu Q., Liu C., Wang Y. (2024). In-plane nonlinear buckling analysis and design method of concrete-filled steel tubular catenary arches. J. Constr. Steel Res..

[6] Liu Z., Zhou J., Xin J., Zhuang Y., Fan Y., Wang K. (2025). In-plane bearing capacity analysis of concrete catenary arch with rigid skeleton: Test and simulation. Adv. Bridge Eng..

[7] Yuan Y., Chen H., Wang J., Wang W., Chen X. (2025). Additive manufacturing of catenary arch structure design: Microstructure, mechanical properties and numerical simulation. J. Mater. Res. Technol..

[8] Fallacara G., Cavaliere I., Melchiorre J., Marano G.C., Manuello A. (2024). Reinterpretation of catenary vaulted spaces: Construction of a prototype and structural evaluation through multibody rope approach. Structures.

[9] Huerta S. (2006). Structural design in the work of Gaudi. Archit. Sci. Rev..

[10] Mishra A.K., Kumar A. (2023). Taking inspiration from medieval architecture: Using catenary arches in place of straight beam as the building block into bending-dominated lattice structures. Eng. Fail. Anal..

[11] Mishra R., Sahani S.K. (2024). Modern designs using parabolic curves as a new paradigm for sophisticated architecture. Asian J. Sci. Technol. Eng. Art.

[12] Li Z., Lee T.-U., Pietroni N., Snooks R., Xie Y.M. (2024). Design and construction of catenary-ruled surfaces. Structures.

[13] Sun B.H. (2022). Small symmetrical deformation and stress analysis of catenary shells of revolution. Acta Mech. Sin..

[14] López R. (2026). A design of an axisymmetric roof with a horizontal rotation axis. Stud. Appl. Math..

[15] Cui G., Cui C. (2025). Shape optimization and mechanical properties analysis of the free-form surfaces. Sci. Rep..

[16] Apellániz D., Vierlinger R. (2022). Enhancing structural design with a parametric FEM toolbox. Steel Constr..

[17] Goldbach A.-K., Lázaro C. (2024). CAD-integrated parametric design and analysis of lightweight shell structures. Structures.

[18] Ağırbaş A., Kutucu S. (2025). Computational design and optimization of discrete shell structures made of equivalent members. Buildings.

[19] Dzwierzynska J., Labuda I. (2021). Modeling of curvilinear steel rod structures based on minimal surfaces. Materials.

[20] Dynamo BIM. https://dynamobim.org/download/.

[21] Rhino3D. https://www.rhino3d.com/.

[22] Dzwierzynska J., Lechwar P. (2024). Comparative analysis of steel bar structures of solar canopies composed of hyperbolic paraboloid units using genetic algorithms. J. Build. Eng..

[23] Dzwierzynska J. (2021). Shaping of curvilinear steel bar structures for variable environmental conditions using genetic algorithms—Moving towards sustainability. Materials.

[24] Dasari S.K., Fantuzzi N., Trovalusci P., Panei R., Pingaro M. (2023). Optimal design of a canopy using parametric structural design and a genetic algorithm. Symmetry.

[25] Cao T., Kotnik T., Schwartz J. (2022). Smooth poly-hypar surface structures: Freeform shells based on combinations of hyperbolic paraboloids. Nexus Netw. J..

[26] Lee C., Shin S., Issa R.R. (2023). Rationalization of free-form architecture using generative and parametric designs. Buildings.

[27] Ajtayné K., Hajdú G., Rodaeva K., Szép J. (2023). Structural optimization of a steel truss for sustainability in parametric environment—A case study. Chem. Eng. Trans..

[28] Goodarzi M., Mohades A., Forghani-Elahabad M. (2021). Improving the gridshells’ regularity by using evolutionary techniques. Mathematics.

[29] Bruno L., Gabriele S., Grande E., Imbimbo M., Laccone F., Marmo F., Mele E., Raffaele L., Tomei V., Venuti F. (2023). Exploring new frontiers in gridshell design: The FreeGrid benchmark. Structures.

[30] Raffaele L., Bruno L., Laccone F., Venuti F., Tomei V. (2024). Holistic performance assessment of gridshells: Methodological framework and applications to steel gridshells. J. Build. Eng..

[31] Fritzsche K., van der Sluis W., Smits E., Bakker J. Capital C: Geometric optimization of a free-form steel gridshell towards planar quadrilateral glass units. Proceedings of the Challenging Glass Conference 7.

[32] Stefanska A., Rokicki W. (2022). Architectural design optimisation in reticulated free-form canopies. Buildings.

[33] Zhong Y., Liu Y., Lu H., Feng R., Xie Y.M. (2025). Design and optimisation of grid shells for maximising both structural performance and panel similarity. Eng. Struct..

[34] Tomei V. (2023). The effect of joint stiffness on optimization design strategies for gridshells: The role of rigid, semi-rigid and hinged joints. Structures.

[35] Yakak B., Atmaca B., Kınalı N.S., Dede T., Grzywinski M., Rao R.V. (2024). Optimization of roofs with solar panels using Rao algorithms. Appl. Soft Comput..

[36] Eigensatz M., Kilian M., Schiftner A., Mitra N., Pottmann H., Pauly M. (2010). Paneling architectural freeform surfaces. ACM Trans. Graph..

[37] Gonzalez Quintial F., Barrallo J., Artiz-Elkarte A. (2015). Freeform surfaces adaptation using developable strips and planar quadrilateral facets. J. Facade Des. Eng..

[38] Smith S., Mansouri I., Garlock M., Wang S. (2024). Predicting maximum deflection of N-edged thin-shelled hyperbolic-paraboloid umbrella using machine learning techniques. Thin-Walled Struct..

[39] Juozapaitis A., Daniūnas A., Ustinovichius L. (2024). Non-linear behaviour and analysis of innovative suspension steel roof structures. Buildings.

[40] Mutovkin A., Diakov S. (2020). Parametric finite element model of the steel frame. Proceedings of EECE 2020: Energy, Environmental and Construction Engineering, St. Petersburg, Russia, 19–20 November 2020.

[41] Su C., Yuan M., Fan Y., Zhu L., Hu N. (2023). Parametric design and modular construction of a large additive-manufactured hypar shell structure. Archit. Intell..

[42] Lapira L., Wadee M.A., Gardner L. (2021). Nonlinear analytical modelling of flat and hyperbolic paraboloidal panels under shear. Proc. R. Soc. A.

[43] S Neogi S.D., Karmakar A., Chakravorty D. (2013). Study of dynamic behavior of multilayered clamped composite skewed hypar shell roofs under impact load. J. Eng..

[44] Das Neogi S., Karmakar A., Chakravorty D. (2017). Finite element analysis of laminated composite skewed hypar shell roof under oblique impact with friction. Procedia Eng..

[45] Neogi S.D., Karmakar A., Chakravorty D. (2022). A study of impact induced stress due low velocity impact on laminated composite skewed hypar shell roof. ASPS Conf. Proc..

[46] Ghosh A., Chakravorty D. (2014). Prediction of progressive failure behaviour of composite skewed hypar shells using finite element method. J. Struct..

[47] Smith S., Wu G., Pawitan K.A., Garlock M. (2022). Feasibility of kinetic umbrellas as deployable flood barriers during landfalling hurricanes. J. Struct. Eng..

[48] Dzwierzynska J., Szewczyk A., Gotkowska E. (2025). Applications of genetic algorithms for designing efficient parking shelters with conoid-shaped roofs. Materials.

[49] He J., Kong F., Huang P., Mei K. (2024). Experimental and numerical investigation on behavior of rectangular closed section steel truss beams with concrete-filled joints. Buildings.

[50] Li N., Cao Z., Bao W., Lin S., Zou T., Yan M. (2024). Experimental study and finite element analysis of heavy-duty escalator truss under full load conditions. Sci. Rep..

[51] Zhou Z., Zhou X., Zhou Q., Fu H., Liu S. (2023). Experimental and numerical study on hysteretic behaviour of a novel frictional energy dissipation steel truss (FED-ST). Materials.

[52] Güldür H., Baran E., Topkaya C. (2021). Experimental and numerical analysis of cold-formed steel floor trusses with concrete-filled compression chord. Eng. Struct..

[53] Quan G., Jamshidi A.R., Nightingale A.P., Trafford A.P. (2022). Experimental and numerical study on the performance of new prefabricated connections for free-form grid structures. Structures.

[54] Autodesk. https://www.autodesk.com/.

[55] (2005). Eurocode 1—Actions on structures—Part 1-3: Snow loads.

[56] (2005). Eurocode 1—Actions on structures—Part 1-4: Wind actions.

